# Transcriptional Profiling of Early Defense Response to White Pine Blister Rust Infection in *Pinus albicaulis* (Whitebark Pine)

**DOI:** 10.3390/genes15050602

**Published:** 2024-05-09

**Authors:** Laura Figueroa-Corona, Kailey Baesen, Akriti Bhattarai, Angelia Kegley, Richard A. Sniezko, Jill Wegrzyn, Amanda R. De La Torre

**Affiliations:** 1 School of Forestry, Northern Arizona University, 200 E. Pine Knoll, Flagstaff, AZ 86011, USAamanda.de-la-torre@nau.edu (A.R.D.L.T.); 2 Department of Ecology and Evolutionary Biology, University of Connecticut, Storrs, CT 06269, USA; 3 USDA Forest Service, Dorena Genetic Resource Center, Cottage Grove, OR 97424, USArichard.sniezko@usda.gov (R.A.S.)

**Keywords:** white pine blister rust, disease resistance, differential expression, transcriptome, whitebark pine, *Pinus albicaulis*

## Abstract

Pathogen perception generates the activation of signal transduction cascades to host defense. White pine blister rust (WPBR) is caused by *Cronartium ribicola* J.C. Fisch and affects a number of species of *Pinus*. One of the most severely affected species is *Pinus albicaulis* Engelm (whitebark pine). WPBR resistance in the species is a polygenic and complex trait that requires an optimized immune response. We identified early responses in 2-year-old seedlings after four days of fungal inoculation and compared the underlying transcriptomic response with that of healthy non-inoculated individuals. A de novo transcriptome assembly was constructed with 56,796 high quality-annotations derived from the needles of susceptible and resistant individuals in a resistant half-sib family. Differential expression analysis identified 599 differentially expressed transcripts, from which 375 were upregulated and 224 were downregulated in the inoculated seedlings. These included components of the initial phase of active responses to abiotic factors and stress regulators, such as those involved in the first steps of flavonoid biosynthesis. Four days after the inoculation, infected individuals showed an overexpression of chitinases, reactive oxygen species (ROS) regulation signaling, and flavonoid intermediates. Our research sheds light on the first stage of infection and emergence of disease symptoms among whitebark pine seedlings. RNA sequencing (RNA-seq) data encoding hypersensitive response, cell wall modification, oxidative regulation signaling, programmed cell death, and plant innate immunity were differentially expressed during the defense response against *C. ribicola*.

## 1. Introduction

The introduction of pathogens represents one of the most challenging threats to natural ecosystems, causing changes in ecological interactions when environmental conditions are variable [1,2,3], exposing native species to new selective pressures, and promoting the activation of defense mechanisms [4,5]. In plants, once a pathogen is detected, a series of signal transduction cascades initiates the host’s defense responses. This defense mechanism involves the hypersensitive response that encompasses direct antimicrobial and antifungal actions and triggers the regulation of programmed cell death at the site of pathogen entry [6,7].

White pine blister rust (WPBR) is caused by the fungal pathogen *Cronartium ribicola* (Basidiomycota, Pucciniales). Native to Asia, the pathogen was accidentally introduced to North America at the beginning of the 20th century by imported seedlings from Europe [8,9]. WPBR affects five-needle pine species (*Pinus* subsection *strobus*); [10], and over 40 susceptible gooseberries species (*Ribes* spp.), causing serious economic and ecological impacts worldwide. As a result of high mortality rates and a reduction in succession events in North American five-needle pines, the survival rate after fungal infection is usually low across species [11]. The host distribution, the lack of connectivity of host populations, and the repeated accidental introductions of the pathogen into northeastern North America have shaped the patterns of dispersal, genetic variation, and the presence of pathogen ecotypes [12,13,14].

Major gene resistance, conferred by a dominant gene with Mendelian segregation, has been documented in four of the nine white pine species native to the U.S.: western white pine (*P. monticola*), southwestern white pine (*P. strobiformis*), sugar pine (*P. lambertiana*), and limber pine (*P. flexilis*). Symptoms of major gene resistance include localized necrosis in infected needle tissue, generally leading to canker-free seedlings [15,16,17,18,19,20]. The genetic management of resistance to white pine blister rust is actively conducted in breeding programs for white pine species in North America, e.g., the National Whitebark Pine Restoration Plan [15,16,17,18,19,20].

Whitebark pine (*Pinus albicaulis*) is a high-elevation five-needle pine (*Pinus* section *strobus*) distributed along southwestern Canada and western United States over soils with coarse talus and exposed bedrock areas deglaciated during the Holocene [21]. It has been classified as endangered in Canada and threatened in the U.S., given disturbances in wildland fire ecology, low succession, climate change, and epidemic diseases. Whitebark pine has been historically affected by multiple epidemic diseases and pests. The most severe is the Mountain pine beetle (*Dendroctonus ponderosae*), which has decimated populations over the Rocky Mountains [20,21,22,23,24]. Whitebark pine is also severely affected by WPBR, with the most susceptible families showing 0% survival after 1–2 years of fungal infection [20]. Major gene resistance has not been documented in the species as only quantitative resistance has been observed as a polygenic trait. Inoculation trials generally affect 100% of seedlings in susceptible and resistant families (offspring of an open-pollinated resistant mother trees) with most showing needle spots, though the number of needle spots can vary. Indeed, 100% of the progeny in the susceptible families develop stem symptoms, in contrast to the infection level in resistant families, which exhibit fewer, latent, or inactive stem symptoms in seedlings [20]. Resistant families can have survival rates of 5–90% five years after WPBR inoculation while the most susceptible families have little or no survival after 3 years [17,20]. Quantitative genetic studies have reported heritability values ranging from 0.23 to 0.92 for WPBR-related traits [20,25,26,27,28,29,30].

In this study, we aim to identify the transcriptional responses in *P. albicaulis* seedlings to WPBR infection addressing the following goals: (1) to characterize the transcriptional response to infection through the identification of the main phytohormonal and metabolic intermediates that constitute the first immune response, and (2) to determine the differentially expressed genes that reflect the defense response after four days of inoculation.

## 2. Materials and Methods

### 2.1. Plant Material

A large-scale WPBR resistance test was set up in 2020 at the USDA Forest Services Dorena Genetic Resource Center (DGRC). In Spring 2020, seeds from 128 parent trees from Oregon and Washington were germinated, then planted, in an amount of one per tube, in 164 cm^3^ Ray Leach containers. Seedlings were grown in unreplicated family blocks in an unheated greenhouse for the 2020 and 2021 growing seasons. Parent tree 06017-004 from the Mt. Hood National Forest was included as one of the resistant checklots. This tree was selected based on its performance in a previous test at DGRC [20]. Prior to inoculation with blister rust, seedlings (up to 60 per family) were placed into a randomized complete block design, with six blocks and up to 10 seedlings per family per block. During the week of September 21, 2021, seedlings were moved into the large fog chamber for inoculation with rust spores. The same inoculation procedure has been used for more than 50 years at DGRC (for details, see Liu and Sniezko, [31]). A heterogenous mix of inoculum was collected from infected leaves of gooseberry plants (the alternate host for *C. ribicola*) with rust at the telia stage from numerous sites in eastern Oregon. The leaves were placed on screens above the pine seedlings under optimum conditions for basidiospores to drop onto the needles, germinate, and infect the seedlings through the stomata. Spore drop was monitored using a series of microscope slides placed throughout the trial. The infected gooseberry leaves were removed once the target spore density was reached, but due to the size of the trial and speed of spore fall, the spore density achieved was higher (average 3773 spores/cm^2^) than the target. Seedlings were left in the chamber for several days to ensure optimal conditions for spore germination and infection of the pine seedlings. Seedlings were transplanted into boxes in the randomized complete block design, with generally 12 families per box and up to 10 trees/family/row, and 10 boxes per block. Phenotypic assessments of rust-resistance-related traits related to needle infection and stem symptoms occurred with two assessments in the first year, and subsequent assessments completed annually in the fall or winter (the final assessment will be completed in fall 2026, five years after inoculation). In the assessments, each seedling was evaluated for the number of needle spots, and the number of stem symptoms, as well as the severity of stem symptoms (0 to 9 scale; 0 = no infection; 1 to 4 = relatively minor impact; 5 to 8 = at least one stem symptom encircling the bole, with increasing vertical expansion from 5 to 8; 9 = dead from rust).

**Figure 1:**
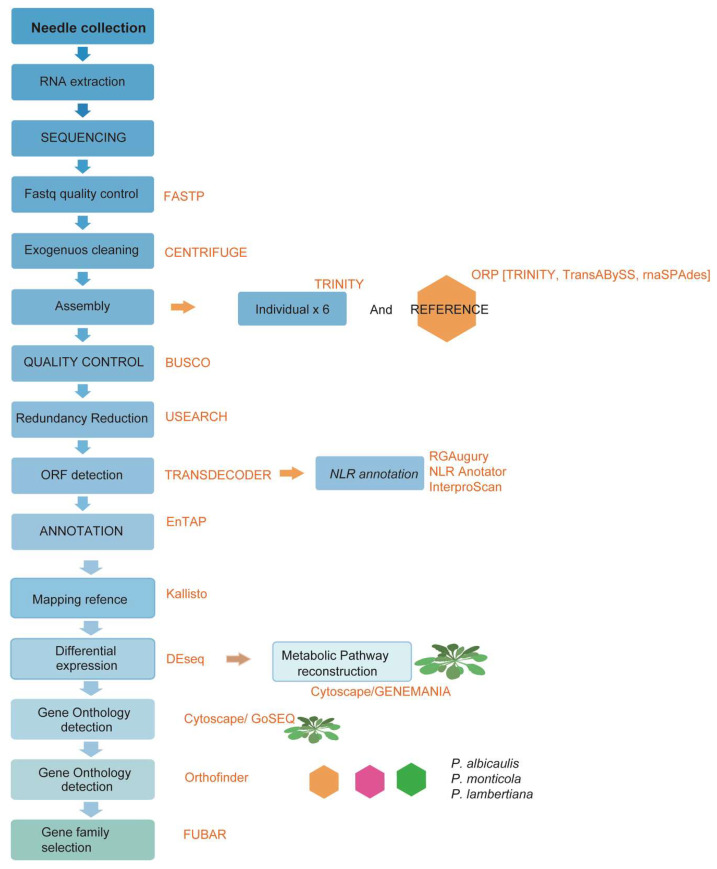
Summary of bioinformatic process for transcriptomic analyses of whitebark pine RNA samples.

For the transcriptomic analysis, needle tissue was collected from six 2-year-old half-sib whitebark pine seedlings (three inoculated and three not inoculated) from parent tree 06017-004. This family had shown quantitative disease resistance in a previous trial [20]. In previous trials of other seedlings from this parent tree, the family showed <60% early stem infection compared with 100% in the most susceptible families. Needle tissue was collected four days after inoculation in September 2021, flash-frozen in liquid nitrogen, and stored at −80 °C until RNA extraction. Figure 1 shows a summary of the bioinformatic processing followed in this study.

### 2.2. RNA Extraction and Sequencing

RNA was extracted using a chemical method (Invitrogen Pure Link Plant RNA Reagent protocol) at the Forest Genomics Lab at NAU. RNA quality was evaluated using Agilent DNF-488 HS Genomic DNA (Agilent, Santa Clara, CA, USA). mRNA library preparation (poly A enrichment) and Illumina NovaSeq 6000 S4 PE 150 sequencing were performed by Novogene (Novogene Corporation Inc., Sacramento, CA, USA).

### 2.3. Transcriptome Assembly

The removal of adapters and quality filtering were performed with fastp (v0.23.2; [32]) to retain a total of 21,131,295 sequence reads (a minimum length of 120 bp; a Phred-scaled quality score of 35) for each individual library. Kraken (v2.0.8-β; [33]) was run using the standard default database in addition to protozoa and fungi sourced from the PlusPF database to taxonomically classify sequencing reads to filter exogenous sequences [34].

To generate a de novo reference transcriptome assembly, the unclassified and trimmed reads from the six libraries were merged and analyzed with Oyster River Protocol (ORP; v2.2.5; [35]), a pipeline that integrates multiple assemblers using the default parameters from Trinity (v2.9.1; [36]), rnaSPAdes (v3.13; [37]), and TransABySS (v2.0.1; [38]). Integrity and quality were evaluated with the single-copy ortholog benchmarking tool BUSCO (v3.0; [39,40]) for the embryophyta and viridiplantae databases (Obd10). Transcript redundancy in the resultant assembly file was reduced by clustering sequences to 90% identity at the nucleotide level in Usearch (v9.0.2132; [41]) and followed by a length threshold via seqtk (https://github.com/lh3/seqtk; accessed on 16 July 2022) to retain transcripts > 300 bp. Transdecoder (v5.5.0; https://github.com/TransDecoder/TransDecoder; accessed on 25 July 2022) was subsequently run to identify coding regions via the detection of ORF within the sequences.

### 2.4. Ortholog Functional Annotation

Annotation of global reference assembly was performed with EnTAP (v0.10.8; [42]) using the sequence similarity search, requiring 80% coverage of both the target and query sequence and an *e*-value of 10^−6^ using Diamond (v0.9.19; [43]) for sequence similarity searches and EggNOG (v4.1; [44]) to provide gene family assignments. Similarity annotation was carried out, aligning against two public databases, NCBI’s RefSeq Complete Protein (v212), and UniProt ([45]; UniProt Consortium 2018), selecting and depurating similarity sequences, with bacteria, fungi, and arthropod matching as potential contaminants. Subsequently, functional annotation involved the assignment of Gene Ontology (GO) terms using Blast2GO (v1.5.5; [46]) and domain identification via InterProScan (v5.25; [47]) with the Pfam-A database (v31.0; [48]). The integrity and quality of the final proteins were reviewed with the single-copy ortholog benchmarking tool, BUSCO (v3.0; [39,40]), using the embryophyta and viridiplantae databases (Obd10).

### 2.5. Differentially Expressed Transcripts

The trimmed paired-end reads from the six libraries were independently mapped to the global transcriptome reference to calculate transcript abundance via Kallisto (v0.44.0; [49]). Mapping rates to the transcriptome reference were higher (71.71% vs. 78.4–81.4%) than those for mapping to the current genome assembly (GCA_034641835.1; [50]). Expression profiles were analyzed by sample using DESeq2 (v1.26; [51]) R package, available in Bioconductor, by removing low-count genes, those below 50 counts, and normalizing them to the median ratio (reads/Kb/mapped) to calculate differential expression. Significantly expressed transcripts were defined at a *p*-adjusted value < 0.1 and a log_2_-fold change > 1.5 to report the differences in samples and detect susceptible changes. Functional descriptions were obtained from the master EnTAP annotation.

### 2.6. Enrichment Analysis

Goseq [52] was used to identify functionally enriched GO terms in differentially expressed upregulated and downregulated genes independently. GO terms of differentially expressed genes were obtained from the EnTAP output, and effective lengths were derived from Kallisto’s output tsv. The results plots were produced by ggplot2, and the number of genes and the log_2_-fold change (*p*-value) of each annotated GO term were described [52,53].

### 2.7. Network Analysis

Cytoscape [54] was used to visualize interactions among DE genes. Genes were matched to putative Arabidopsis orthologs assigned through EnTAP, integrated with the GeneMANIA plugin (v3.5.2; [55]). These interactions were further enriched by considering the abundance of GO terms in the ClueGO plugin (v2.5.9; [56]). Settings allowed for every interaction with automatic weighting at a maximum of 20 resultant genes.

The analysis was subsequently repeated using differentially expressed genes from the inoculated samples, aiming to construct a classified comparative network. The analysis was focused on the differentially expressed genes and their interacting genes within the stress category (phytohormones, immune responses, chitinase activity, and oxidative stress reduction). Leveraging the differentially expressed genes and their KEGG KO identifiers, the metabolic pathway was reconstructed with the BlastKoala tool ([57,58]; https://www.genome.jp/kegg-bin/show_pathway?map00941, accessed on 1 April 2024). This approach considered both the presence and absence of intermediates within these pathways.

### 2.8. NLR Genes Annotation

InterProScan (v5.35-74.0; [47]) was used to scan the translated protein sequences for the identification of the functional domains using the following databases: Pfam (v32.0), SMART (v7.1), CDD (v3.16), Gene3D (v4.2.0), PRINTS (v42.0), and SUPERFAMILY (v1.75). These protein domain annotations were used to determine the NLR architecture and classify the transcripts into NLR subfamilies. The protein sequences were also assessed with the RGAugury (v1.0; [59]) pipeline, which uses ‘Blast similarity searches’ to filter potential resistance genes before applying InterProScan. Finally, NLRannotator [60] was used to scan the coding sequences of each transcript for NLR-associated motifs, and these motifs were combined and used to identify the domains and subfamilies of each potential NLR. The results of these three methods were combined, and ambiguous annotations were filtered out to generate a more complete set of NLRs.

### 2.9. Response to Infection across White Pine Species

Differences in gene family abundance among inoculated and non-inoculated whitebark samples were assessed by comparing gene families from individual transcriptome assemblies with Orthofinder (v2.4; [61]). Common transcripts present in all whitebark pine individuals were compared between treatments. Finally, a third comparison was implemented using the gene families’ consensus cluster of inoculated samples to compare with transcriptomes constructed with infected tissue in *P. lambertiana* (PRJNA949211; unpublish data) and *P. monticola* PRJNA222839; [62]). The data for these white pines were obtained from the NCBI database and were used to identify orthologous gene signaling across different levels of resistance and susceptibility across species.

Signatures of selection were estimated across the 25 most abundant gene families in the three species of white pine based on the Orthofinder output. FUBAR (fast unconstrained Bayesian approximation; [63]) was used to infer nonsynonymous (dN) and synonymous (dS) rates with a cut off using the posterior probability > 0.98 and a Bayes factor > 100 as candidates for selection. FUBAR reports posterior probabilities from 0 to 1, with values > 0.9, strongly suggesting positive selection [63]. To run FUBAR, a nucleotide codon alignment was produced for each gene family sequence. Bayesian probability on a per-site basis for every given coding alignment and the corresponding species gene tree were generated by Orthofinder [61].

## 3. Results

### 3.1. Quantitative Resistance Response

**Figure 2:**
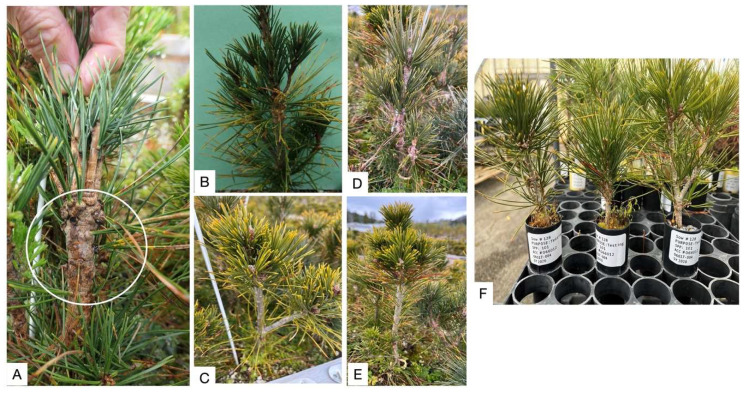
Phenotypical characterization of WPBR. (**A**). Canker formation in seedling 20 months after the inoculation (white circle). (**B**) Needle spot and leaf discoloration one year after the inoculation. (**C**). Inoculated seedling (Fam: 06017-004; ind: 1281) 18 months after inoculation. (**D**). Inoculated seedling (Fam: 06017-004; ind: 1282) 18 months after inoculation. (**E**). Inoculated seedling (Fam: 06017-004; ind: 1283) 18 months after inoculation. (**F**). Non-inoculated seedlings (Fam: 06017-004; ind: 1284, 1285, and 1286) after 18 months of the experiment.

White pine blister rust inoculation was very successful, with 99.3% of the seedlings in the 128 families showing needle spots at first assessment, and 65.3% of the seedlings showing stem symptoms by the 2nd assessment (family means varied from 11.6 to 100%, with both susceptible and control families having seedlings with stem symptoms). The three inoculated individuals (from which RNA was extracted) had at least 13, 14 and 25 needle spots, nine months after inoculation (Spring 2022), and after 13 months (Fall 2023), this family had 55% of seedlings showing one or more stem symptoms (including two of the sampled seedlings). Figure 2 shows the appearances of needle spots and cankers, and the differences between the seedlings used in this study compared with the non-inoculated samples, 18 months after the inoculation.

### 3.2. Transcriptome Assembly and Annotation

Illumina NovaSeq 6000 S4 PE 150 sequencing produced an average of 23,984,843 reads per library. After quality trimming, an average of 21,865,768 reads per library were kept for further analyses (Table S1). The reference de novo assembly produced 139,837 putative transcripts with an N50 of 2882 bp (Table S2). Initial filtering consisting of redundancy reduction, length threshold, and frame selection reduced this set to 66,233 sequences. Additional filtering removed 1072 sequences as potential contaminants based on a similarity search against fungus species (Table S3). From the 65,161 transcripts set with a high-quality sequence similarity search alignment (NCBI Plant RefSeq), 43,180 (65.19%) received primary annotations from *Amborella trichopoda*, *Juglans microcarpa*, *Nymphaea colorata*, and *Dioscorea cayenensis*. The total annotated unique sequences including the gene family and/or sequence similarity search was 56,796 (85.6%; Table S4). Single BUSCO completeness was evaluated at 88.4% for embryophyta and 95.3% for viridiplantae database lineages of OrthoDB (v10; [39,40]).

### 3.3. Differential Expression Analyses

**Figure 3:**
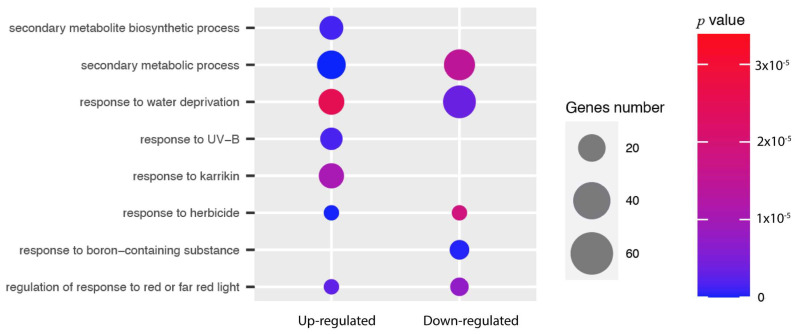
Biological process gene ontology (GO) enrichment analysis. This figure shows the eighth most enriched GO terms of upregulated (3 × 10^−5^) and downregulated (0) genes in *P. albicaulis* related to the abiotic stress response.

The six libraries were processed independently via Kallisto [49]. Mapping against the assembled reference was performed using settings for 100 bootstraps and 8 threads. Mapping rates ranged from 78.4% to 81.4%. In total, 599 transcripts were differentially expressed, from which 375 were upregulated and 224 were downregulated in the inoculated samples (Figure S1). The differential expression of transcripts showed high heterogeneity among samples (Figure S2). Chalcone synthase was the most upregulated (3.95 log_2_-fold change) among samples. Similarly, differences in the overexpression of orthologs were also found in abscisic stress-ripening protein 2 (3.5 log_2_-fold change), and in Leaf Rust 10 (Lr10), plus a 431 disease resistance locus receptor-like protein kinase-like 1.2 (3.6 log_2_-fold change). In the non-inoculated samples, the most significantly downregulated transcript was the transcript that translates into Calmodulin-binding protein (−12.76 log_2_-fold change). Gene enrichment analysis (Figure 3 and Figure S3) showed that upregulated transcripts had an over-representation of genes involved in secondary metabolism and flavonoid metabolic processes (GO:0009812) and the response to karrikin (GO: 0080167), while the downregulated transcripts were enriched in water deprivation response process.

### 3.4. NBL Expression after Inoculation

**Figure 4:**
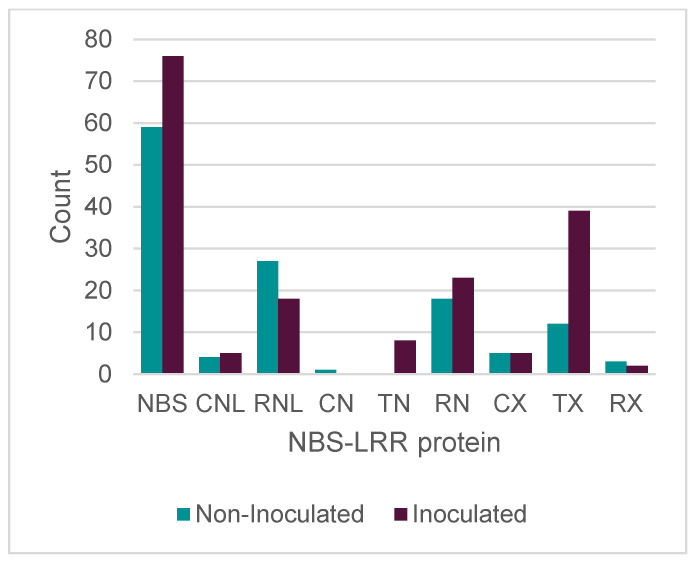
NBS-LRR protein comparison between non-inoculated and inoculated samples of *P. albicaulis*. The classification was based on the identified domains. NBS (nucleotide-binding site (NB-ARC)); CNL (N-terminal coiled-coil nucleotide-binding site and Leucine domain (CC)(NB-ARC)(LRR); RNL, resistance to powdery mildew8 (RPW8) (NB-ARC) (LRR); NL, nucleotide-binding site and Leucine domain (NB-ARC) (LRR); CN, N-terminal coiled-coil domain and nucleotide-binding site (CC) (NB- ARC); TN, Toll/IL-1 (TIR) (NB-ARC); RN, resistance to powdery mildew8 and nucleotide-binding site (RPW8)(NB-ARC); CX, coiled-coil domain (CC); TX, Toll/IL-1 (TIR); RX, resistance to powdery mildew8 (RPW8).

The nucleotide-binding domain leucine-rich repeat (NLR) gene family represents one of the most important families of disease resistance genes in plants, with many genes recognized as potential candidates for the major resistance gene as well as important intermediates in quantitative resistance against WBPR [14,64]. Results of this study found a nucleotide-binding site leucine-rich repeat (NBS-LRR) increase from 59 transcripts in the non-inoculated samples to 76 transcripts in the inoculated samples. Factors involved in the signal transduction to immune plant defense, like TIR-X or TX (14:39) and RN (18:25), were more frequently observed than changes in TNL or CNL, which are more frequently involved in the recognition of the pathogen. In contrast, the proteins NL showed a reduction (28:19) compared with the inoculated samples (Figure 4).

### 3.5. Gene Interactions

**Figure 5:**
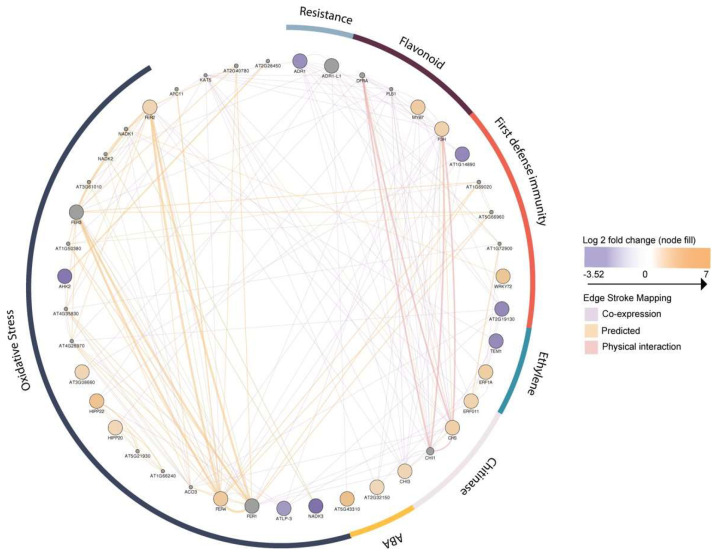
*P. albicaulis* gene interaction classification: upregulated in orange circles and downregulated in purple circles in the inoculated samples, dividing genes according to stress functionality. Gray circles represent interactor genes viewed with GeneMANIA-related genes. Crossing lines yielding co-expression interactions are shown in purple, and shared protein domains are shown in teal.

The networks of differentially expressed genes were constructed with the 272 Arabidopsis orthologs involved in constitutive metabolism (carbohydrate, amino acid, lipid metabolism, and signal transduction), chloroplast (energy metabolism), developmental (cell growth and death), membrane (Glycan biosynthesis and metabolism), stress (biosynthesis of secondary metabolites), and transport activities. The classification was based on KO annotation and function (Figures S4 and S5). Within the stress regulation group, the activities of several phytohormones were reconstructed to better represent the ethylene and flavonoid interactions, and the activities of chitinases (Figure 5).

**Figure 6:**
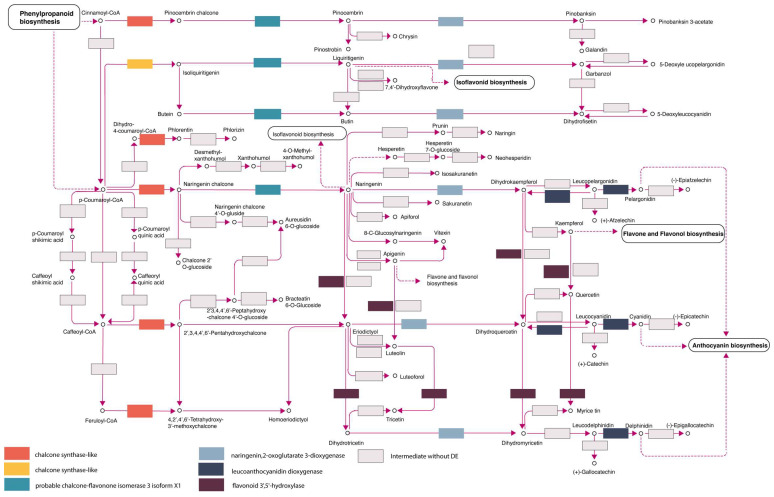
Reconstructed flavonoid pathway. Modified from KEGG ID: map0941. Boxes represent the protein intermediate, and colored intermediates represent the proteins with differential expression in *P. albicaulis* after fungal inoculation.

From the 980 GO terms represented in the DE transcripts, 226 entries were defined based on their KO term (KEGG Ontology). The most complete reconstructions were obtained from the biosynthesis of secondary metabolites such as flavonoid biosynthesis, intermediates that regulate the phenylpropanoid biosynthesis, flavone and flavonol biosynthesis, and anthocyanin regulation (Figure 6; KEGG ID:00941; [65]).

### 3.6. Gene Family Analysis

De novo assemblies conducted by Trinity [36] generated between 34,763 and 40,849 transcripts. The assemblies were processed via USEARCH v9.0.2132 to reduce the redundancy of transcripts within each assembly (0.9 similarity index). Local clustered assemblies were frame-selected using Transdecoder v3.0.1 to identify the longest and most likely open reading frames. Between 39,359 and 52,025 transcripts were retained for each library (Table S2).

The individual assemblies had an average of 251 potential contaminants from species such as *Schizosaccharomyces pombe*, *Saccharomyces cerevisiae*, and *Bacillus subtilis* (Table S3). These contaminants were filtered out from the dataset. Out of the total transcripts, 14,631 (comprising 61.13%) were annotated through a sequence similarity search using NCBI Plant RefSeq. A final set of 19,047 sequences were annotated via gene family analysis or a sequence similarity search, representing 79.6% of the total dataset. The proportions of annotated sets with a single completeness BUSCO (Benchmarking Universal Single-Copy Ortholog) were 75.9% for embryophyta and 79.9% for viridiplantae databases lineages of OrthoDBv10 ([39,40]; Table S2).

**Figure 7:**
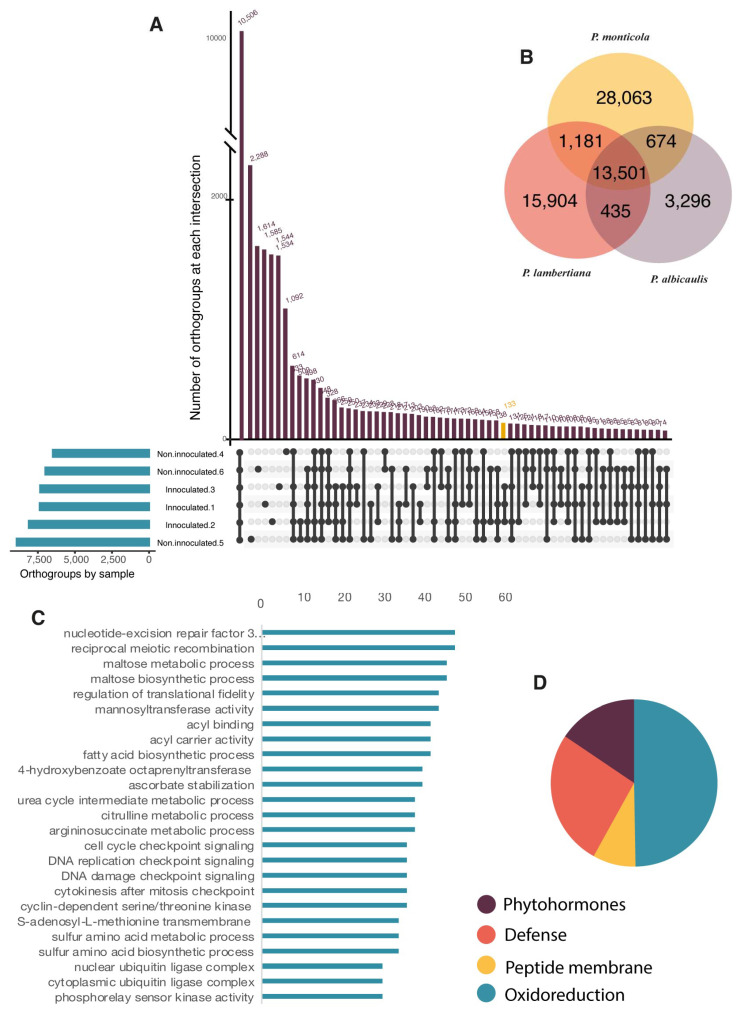
Gene family comparison. (**A**) Filtered gene families shared among six *Pinus albicaulis* transcriptomes identified by Orthofinder. (**B**) Venn diagram depicting the unique and shared gene families in the protein comparison among transcriptome assemblies for *P. albicaulis* (blue), *P. monticola* (yellow), and *P. lambertiana* (orange). (**C**) Abundance of the 25 most represented orthogroups on WPBR-infected transcriptomes across *P. albicaulis, P. lambertiana*, and *P. monticola*. (**D**) Gene abundance proportion for orthogroups shared across the three species activities related to oxidoreduction (blue; 378), peptide membrane constitution (yellow; 64), defense (orange; 200), and phytohormones (purple; 116).

Comparison among the six annotated Whitebark pine transcriptomes, representing 257,144 of the 258,286 genes, were assigned to 30,705 orthologous gene families (Figure 7A). Of these, 10,506 orthogroups (34.2%) were observed in all samples and 2,1024 orthogroups (8.1%) were specific to a single sample. No single-copy orthogroups were identified, and 1142 were unassigned genes (0.4%). The comparison between treatments identified 20,033 orthogroups that were unique to the inoculated samples and 20,072 orthogroups in the non-inoculated individuals (Figure 7A). The inoculated orthologous gene families showed increased expression activity, with 78 genes involved in secondary metabolite processing, such as that of prenol (GO:0016092), isoprenoid (GO:0016097), monoterpenoid (GO:0016099), carotene (GO:0016119), sterol (GO:0016127), brassinosteroid (GO: 0016131, GO:0016133), and saponin (GO:0016136; Figure 7B).

### 3.7. Regulation of Multiple TF Families in the WPBR Pathosystem

Transcription factors (TFs) play diverse roles in plant development and response to stress, and function as the main triggers to regulate gene expression. Our differential expression analyses found three major TF families involved in plant defense response: MYB, bZIP, and WRKY [66]. MYB TFs were consistently upregulated (MYB1-like, transcription factor TRY-like (2.1 log_2_-fold change), and MYB-related protein Hv1 (3.8 log_2_-fold change). Similarly, there were 11 ethylene-dependent transcription factors, and the upregulation of 2 chitinase regulators was shown. WRKY TFs found in this study were downregulated for transcription factor 72B-like (−7.08 log_2_-fold change) and upregulated for WRKY transcription factor 6-like (7.3 log_2_-fold change). Both transcription factors have been described as activators of salicylic acid (SA) and jasmonic acid (JA) in the abiotic response to salt and drought stress [67].

### 3.8. Conservative Response among White Pine Species

To compare and recognize the common components through WPBR infection across white pine species, we compared complete transcriptomes of *P. monticola* and *P. lambertiana* with the *P. albicaulis* transcriptome developed in this study. All transcriptomes were developed from plants inoculated with *C. ribicola*. Given the heterogeneity across sampling tissues and the experimental conditions, we decided to carry out this analysis in the complete transcriptome at gene family level. Previously reported DE genes in *P. monticola* and *P. lambertiana* were not included in this analysis. A total of 215, 849 of the 233,951 genes were assigned to 39,879 orthologous gene families across the species comparison (Figure 7B). From these, 13,501 orthogroups (33.8%) were observed in all the white pines and no single-copy orthogroups were identified, while 18,102 were genes that were not assigned to any orthogroup (7.7%).

In the comparison across species, the most abundant gene families were DNA and RNA damage and repair machinery, cell division, replication, and transcription intermediates, and MAP kinase regulation, among others. In the comparative analysis of expressed gene families, 200 orthogroups were found to be involved in toxin reduction and immune response, 116 were found to be involved in phytohormone synthesis, and 93 were osmoprotectants and oxidoreduction synthesis factors (Figure 7C,D). Variations in the sample conditions, times, tissues, and ages limited this comparison to the shared orthologs among the three species. A codon-level analysis of positive natural selection was performed with FUBAR [68]. A comparison of twenty-five gene families across species found that the mannosyl transferase family showed signatures of positive selection (0.98 Prob[α < β]).

## 4. Discussion

### 4.1. The Whitebark Pine Initial Defense Response at the RNA Level

Defense and disease resistance traits involve intricate responses spanning multiple physiological and regulatory levels. While there are some reports on the hypersensitive response and the avirulence factors in resistant phenotypes [18,69], the quantitative resistance response in the initial phase of infection in Whitebark pine has been not characterized. This study shows the activation of mechanisms that manifest during the initial phase of defense. Specifically, it underscores the overexpression of orthologs of Chalcone synthase, which emerged as the most significantly upregulated gene (with a 3.95 log_2_-fold change). This gene is closely linked to the production of chalcones, which is crucial in the early stages of flavonoid biosynthesis, and with a pivotal role in defense mechanisms [6]. Our results indicate the overexpression of abscisic stress-ripening protein 2 (3.5 log_2_-fold change). ASR genes have been described as promotors of abscisic acid during salinity and drought abiotic stress responses or sugar metabolism. Overexpression of the LLA23 gene in Arabidopsis conferred cold and freezing tolerance [70], while other members like MpASR in maize, ZmASR3 in Arabidopsis, or BdASR1 in wheat increased tolerance to biotic stress and drought [71,72].

The upregulation of the enhanced disease resistance 2 isoform X3 (2.34 log_2_-fold change) was detected in our study, and orthologs of this gene have been reported to regulate the early interaction with the pathogen and to trigger programmed cell death in *Elaeis guineensis* infected by *Ganoderma boninense* [73,74].

Our findings also pointed towards the upregulation of a PR5 protein family member that halts assembly and enhances fungal cell wall permeability [75,76]. Wang [77] revealed the early recognition of the rust pathogen12 h post-inoculation in wheat plants. Among the downregulated factors important to describing the progress, contention, and regulation of infection was the ortholog CRJ31_CRYJA pathogenesis-related thaumatin-like protein 3.1 (8.32 log_2_-fold change). An ortholog of this gene was found to be involved in SA, JA, and abscisic acid (ABA) synthesis under biotic and abiotic stress stimuli [74].

### 4.2. Role of NLR Genes in Disease Response in Whitebark Pine

This study has identified 20 potential NLR transcripts in the inoculated sample. NLRs consist of an N-terminal domain, a conserved nucleotide-binding domain (NB-ARC), and a leucine-rich repeat (LRR) domain, which can be used to identify potential NLRs from transcripts or gene sequences. A search for the three major subfamilies distinguished NLRs by their N-terminal domains; those with a TIR domain are TNLs, those with a coiled-coil domain are CNLs, and those with an RPW8 domain are RNLs [78,79]. There were no complete TNL representatives, and seven partial TNL transcripts were only identified in the inoculated samples. TNLs are most abundant NLRs in conifers, and the fact that so few could be identified in this study might suggest that our set of annotated NLRs was not complete. However, these results were very fragmented, and more than half of the potential NLRs were identified only by their NBARC domain. Only about 20–25% of the identified NLRs in both samples could be classified, meaning that almost three-fourths of the potential NLRs lacked an N-terminal domain. For the few that could be classified, the subfamily ratios were not representative of the ratio of TNLs, CNLs, and RNLs in conifers [79].

### 4.3. The Interaction between Ethylene and Chitinase Activity as Response to WPBR

Our results indicated the downregulation of 11 ethylene-dependent transcription factors and the upregulation of two chitinase regulators, reflecting the first stages of plant defense through fungal infection. TFs have been reported during two important events: (1) in the first steps of germination, promoting cell differentiation on the meristem tissue [67]; (2) during flavonoid biosynthesis, as intermediates of phenolic compounds (chalcones, flavones, and flavonones) [80] induced by rust infection [62]. The regulation of chitinases degrade chitin, the component of the fungal cell wall [6]. Plant chitinases belong to family 19 of glycosyl hydrolases and play a significant role in embryogenesis and ethylene synthesis, in which they are regulated by a feedback loop induced by ethylene [81]. Their signaling has been widely studied to determine the changes between the hypersensitive response and the starting of programmed cell death [82], through the induction of pathogenesis-related genes or Leaf Rust 10 (Lr10) genes (e.g., *Puccinia triticina* fungus infection in wheat). Our study found an upregulation of Lr10, a disease-resistance locus receptor-like protein kinase-like 1.2 (6.3 log_2_-fold change) whose orthologs have been reported to confer enhanced resistance to leaf rust to prevent rust sporulation for *P. triticina* and *Puccinia malvacearum* [83,84].

Chitinases with a structural chitin-binding region and a catalytic domain (Class IV) have been previously correlated with increased quantitative resistance to *C. ribicola* in *P. monticola* and *P. albicaulis* [31,62]. Peery [85] reported an upregulated signal on *P. contorta* and *P. banksiana* inoculated with *C. harknessii*. The interaction between ethylene and chitinase signaling is expressed constitutively at a low level [82]. Chitinases’ interaction with WRKY transcription factors during the ABA response produced an increment in drought tolerance in *P. pinaster* and *P. pinea* [86]. Keefe [87] and Mauch [88] reported that chitinase accumulates around wound tissue from 5–20 min to 28 days post-infection on bean and tobacco leaves treated with ethylene, slowing down pathogen growth and decreasing sporulation at later stages of fungal infection.

### 4.4. Role of Secondary Metabolites in WPBR Defense: The Flavonoid Pathway

The proportion of enriched gene families and differential expression changes detected in this study highlight the active role of secondary metabolites in the defense system of Whitebark pine. Secondary metabolites, such as flavonoid intermediates, play a significant function in redox regulation and as modulators of mycotoxin and antimicrobial compounds. At the beginning of pathogen invasion, a hypersensitive response induces reactive oxygen species (ROS) production [6]. The accumulation of ROS induces programmed cell death but is regulated by the effect of flavonoids [88,89]. The upregulation of flavonoid hydrolase family proteins found in this study (Figure 6) has been previously documented in biotic and abiotic stress and as a regulator of phytohormones like ABA and JA [90]. Liu [62] induced the biosynthesis of secondary metabolism with methyl jasmonate to enhance the defense response in *P. albicaulis* infected by *C. ribicola*, showing an association between the expression levels of hydroxylase genes and the stress exposure.

The synthesis of flavonoids according to the differential expression changes detected in our study corresponded to several levels of the production of flavones, flavanones, dihydroflavonols, and flavonols with the upregulation of FL3H1CROXC flavanone 3-dioxygenase (7.45 log_2_-fold change), flavonoid 3’,5’-hydroxylase 2-like (8.9 log_2_-fold change), and chalcone-flavonone isomerase 3 (3.21 log_2_-fold change). In addition to this, the upregulation of MYB transcription factors further supports the notion that most intermediates within the entire flavonoid biosynthesis pathway exhibit high levels of expression.

The observable changes in phenotype during the infection assessments after *C. ribicola* inoculation are associated with the accumulation of pigments derived from flavonoids, specifically an increase in anthocyanin and flavonol levels in both rust spots and discolored tissues in susceptible or resistant phenotypes [91,92,93]. Our findings revealed a progressive increase in the appearance of these pigments over time, representing an easily observed defense mechanism showing the severity of the symptoms in the resistant individuals.

### 4.5. Comparisons across White Pine Species

The characterization of disease resistance in white pines presented here involves a broad-range comparison across various experimental designs. These designs often reduce the resolution needed to compare differential expression or gene-by-gene interactions across species. Nonetheless, comparing orthologs offers a common basis across species with diverse strategies and susceptibility ranges. However, several challenges persist: First, phenotypic trials represent a long-term, time-consuming, and labor-intensive commitment, as the impact of infection on survival varies from plant to plant and could potentially be overestimated due to slow rusting and delayed symptom development [94]. Second, in the absence of white pines’ genomic resources, transcriptome comparative approaches remain the best way to identify the conserved WPBR defense-related gene families. WPBR has infected North American forests in recent times, affecting a few cohorts of generations. The comparison of white pine species represents an effort to recognize the infection as a form of ongoing selective pressure and to address the adaptive potential in natural populations. The gene families’ comparison among *P. lambertiana* (PRJNA949211; unpublished data), *P. albicaulis*, and *P. monticola* (PRJNA222839; [62]) showed similar mechanisms that included response to stress, pathogen detection, necrosis of infected cells, response to oxidative stress, and immune effector processes that are highly regulated via the early induction of defense-related genes and complex phytohormone signaling, including SA, JA, and ethylene mechanisms.

The abundance of transcript intermediates in flavonoid and anthocyanin production involved in the defense response found in this study suggests the activation of the immune response, as previously found in *Pinus pinaster* infections, to other fungal pathogens such as *Fusarium circinatum* or *F. oxysporum* [95]. These metabolites were also found to be associated with abiotic stress in other pine species such as *P. pinea* through oxidative stress [96] or *P. halepensis* during water deficiency [97]. This study points to a convergent strategy between species during pathogen recognition.

### 4.6. Other Aspects to Consider When Studying WPBR Infection in Whitebark Pine

The studies of quantitative resistance in whitebark pine need to consider the climatic and ecological variance between distribution, humidity, and temperature during inoculation, as all these factors have been reported to increase disease susceptibility [98]. For example, [14,17] reported a cline with more resistant phenotypes in colder locations. In contrast, King [99] and Shanahan [100] suggested that the level of infection has a relationship with the regional abundance of gooseberry species and the presence of other host species.

In this in vitro experiment, it is important to note that while closely related individuals were cultivated under controlled inoculation conditions, transcriptomic experimental design and phenotypic variation pointed towards differences in symptom intensity, reflecting the intricate interplay of genetic, environmental, and physiological factors at play. These differences were noticeable, given the inherent characteristics of the whitebark pine’s quantitative response. In the absence of a major gene for resistance, the quantitative response is likely to be a consequence of the small effects of many genes segregating for resistance, as was observed in other white pine species [18]. This polygenic control might have contributed to the high heterogeneity observed among samples in their response to infection.

Similar previous transcriptome studies by Bair [101] and Liu [62] detected a pattern of upregulation in a variety of pathogenesis-related proteins in a comparison between inoculated and non-inoculated susceptible vs. resistant seedlings. Hoff [102] and Sniezko [20] suggested that some symptoms such as bark reactions have positive associations with an increase in survival and could be used as indicators of quantitative response. Variations in the timing of emergence of stem symptoms have also been previously observed between resistant and susceptible phenotypes.

The broad ranges of transcriptional phenotypes in the characterization of disease resistance are controlled by complex regulatory networks. This study included a small number of inoculated individuals to explore the transcriptomic response, in one resistant family, to white pine blister rust. The main potential limitations of this study were the low resolution for detecting the effect of time over the progression of infection and an incomplete overlap between the expression profiles across the samples processed, which was unintended but inevitable due to many causes such as the quantitative defense, quality control, and the lower number of replicates used in this study. Nevertheless, the contrast between inoculated and non-inoculated samples found in this study showed the rapid activation and regulation of intermediates in the defense response after pathogen detection.

## 5. Conclusions

Transcriptomic profiling from *P. albicaulis* needles constitutes an initial understanding of the quantitative defense response to *C. ribicola* infection. We were able to recognize the first signals of infection to describe the defense strategy in seedlings of a resistant half-sib family. The RNA-seq data analysis conducted four days post-inoculation unveiled the early hypersensitive response. This response was characterized by several key events, including the modification of the cell wall, where chitinases were markedly overexpressed to degrade chitin, inadvertently facilitating pathogen invasion. Additionally, there was a well-regulated signaling pathway for ROS, and the presence of flavonoid intermediates played a pivotal role in redox regulation.

Later reactions to pathogen infection are important to consider in future research projects. These projects should include multiple sampling times post-inoculation to recognize the breaking point in quantitative response in resistant phenotypes. Given the genetic variance found in this study, population level studies of susceptibility and defense mechanisms will be required to inform reforestation, management, and conservation strategies.

## Data Availability

The RNA reads and the de novo Transcriptome assemblies are available in NCBI via Bioproject PRJNA933606. The code used is available at: https://gitlab.com/lcorona/wbp_transcriptomics (accessed on 20 September 2022).

## Supplementary Materials

The following supporting information can be downloaded at: https://www.mdpi.com/article/10.3390/genes15050602/s1, Figure S1. Heterogeneity expression patters in *P. albicaulis*, comparing inoculated vs. noninoculated individuals; Figure S2. Volcano plot for differentially expressed genes of *P. albicaulis*, obtained by comparing inoculated vs. non-inoculated individuals; Figure S3. Molecular function gene ontology (GO) enrichment analysis; Figure S4. Interaction network for *P. albicaulis* gene interaction classification; Figure S5. Gene interaction based the ortholog classification of DE gene families associated with abiotic response. Table S1: Statistics number of reads per sample and aligned to the reference transcriptome for each sample; Table S2. Summary statistics for de novo assembly; Table S3. Contaminants detected based on similarly search annotation; Table S4. Annotation statistics. Table S5. Transcriptome details of White pine species with transcriptomic evidence of infection of WBPR used in this study.

## Abbreviations

ABA	Abscisic acid
bZIP	Basic region/leucine zipper motif
DE	Differentially expressed
DGRC	Genetic Resource Center
HR	Hypersensitive response
PR	Pathogenesis-related protein
PCD	Programmed cell death
JA	Jasmonic acid
LRR	Leucine-rich repeat
MYB	Myeloblastosis transcription factor
NLR	Nucleotide-binding domain leucine-rich repeat
NBS	Nucleotide-binding site
ROS	Reactive oxygen species
SA	Salicylic acid
TF	Transcription factor
WPBR	White pine blister rust
