# Supplementary material for: Transcriptional Profiling of Early Defense Response to White Pine Blister Rust Infection in Pinus albicaulis (Whitebark Pine)
Source: Genes (Basel). 2024 May 9;15(5):602. doi: 10.3390/genes15050602 (PMC11121556; doi:10.3390/genes15050602)
Supplement: Supplementary file 1 [file genes-15-00602-s001.zip › genes-2971427-supplementary.pdf]

Supplementary materials

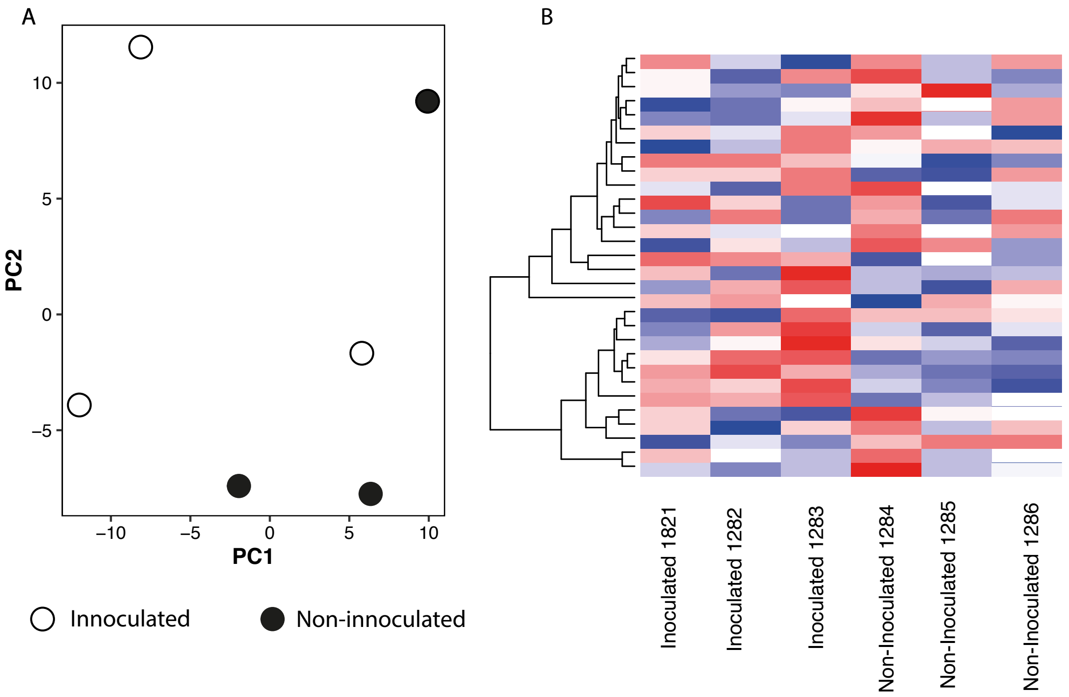

Figure S1. Heterogeneity expression patters in *P. albicaulis*, comparing inoculated vs. non-inoculated individuals. A. PCA constructed by 599 differential expressed genes. B. Heatmap clustering 30 most DE genes.

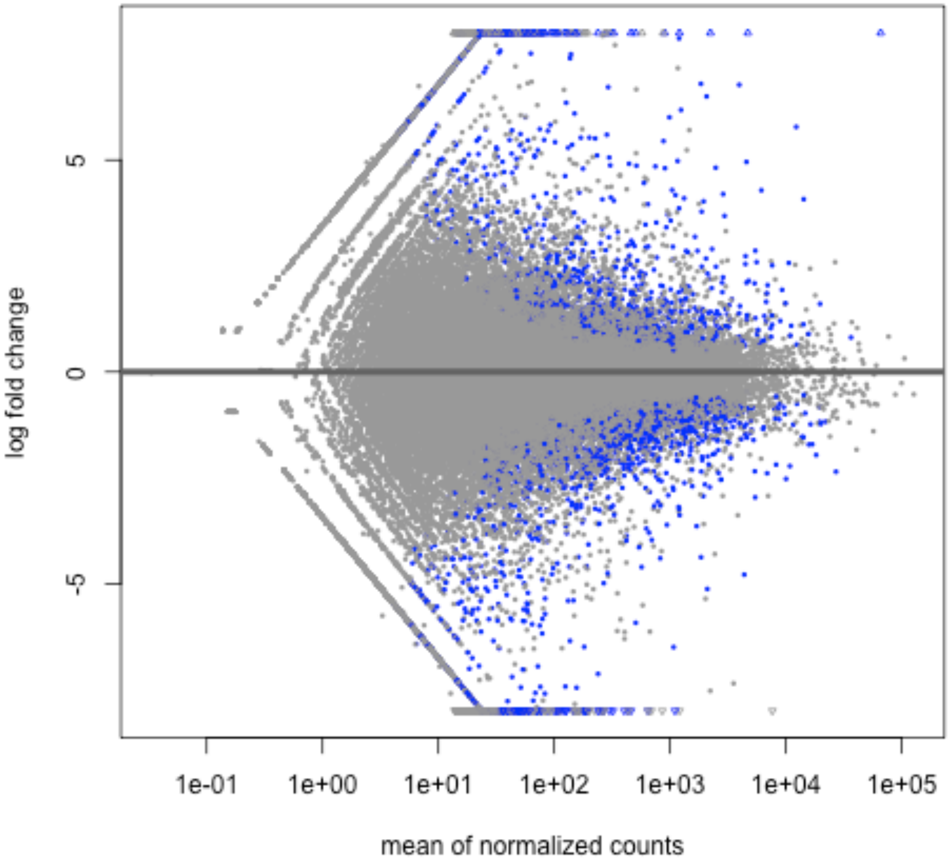

Figure S2. Volcano plot for differentially expressed genes of *P. albicaulis*, obtained by comparing inoculated vs. non-inoculated individuals.

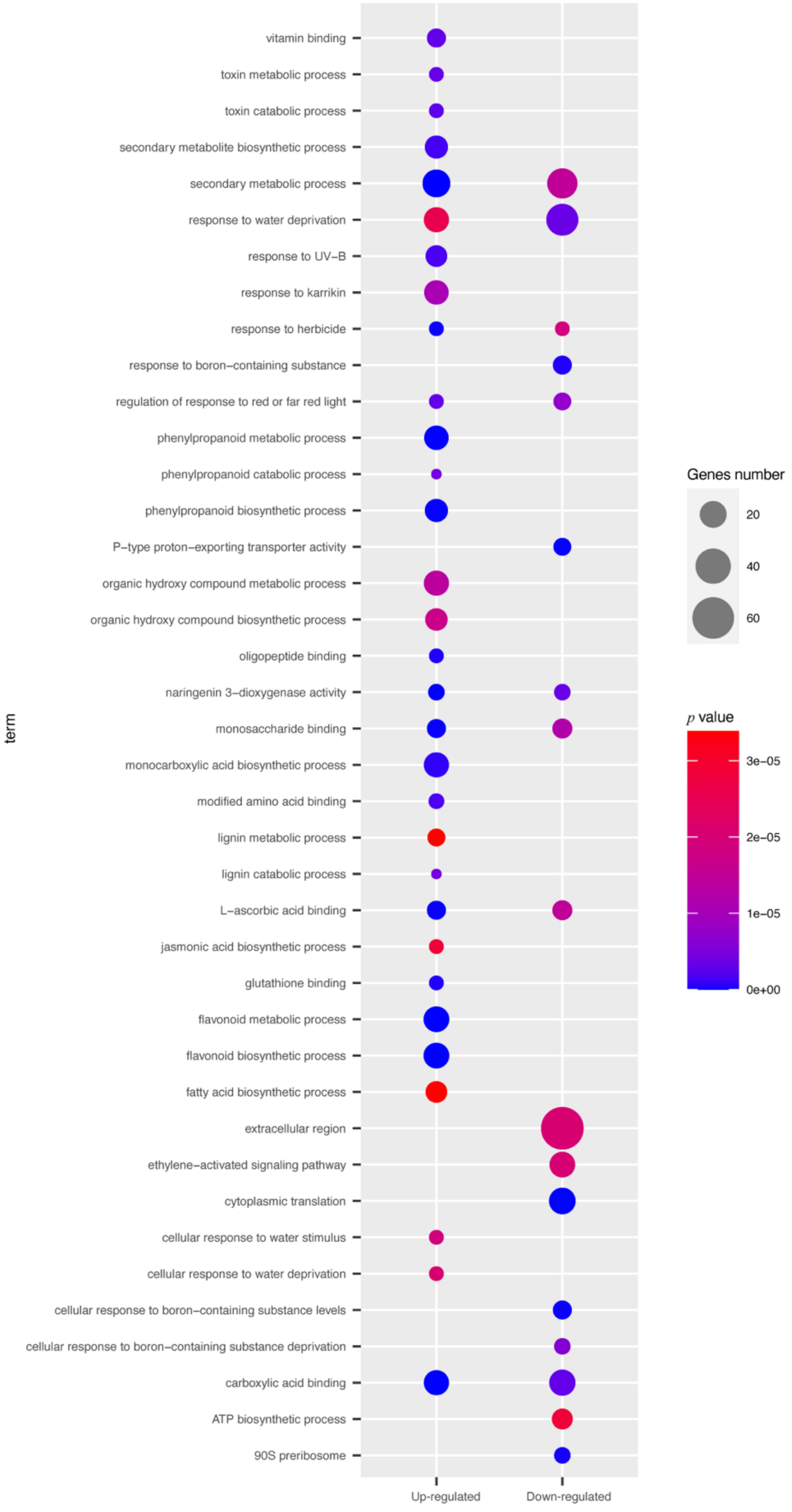

Figure S3. Molecular function gene ontology (GO) enrichment analysis. Enriched GO terms of up-(3e^{-5}) and down-(0) regulated genes in *P. albicaulis.*

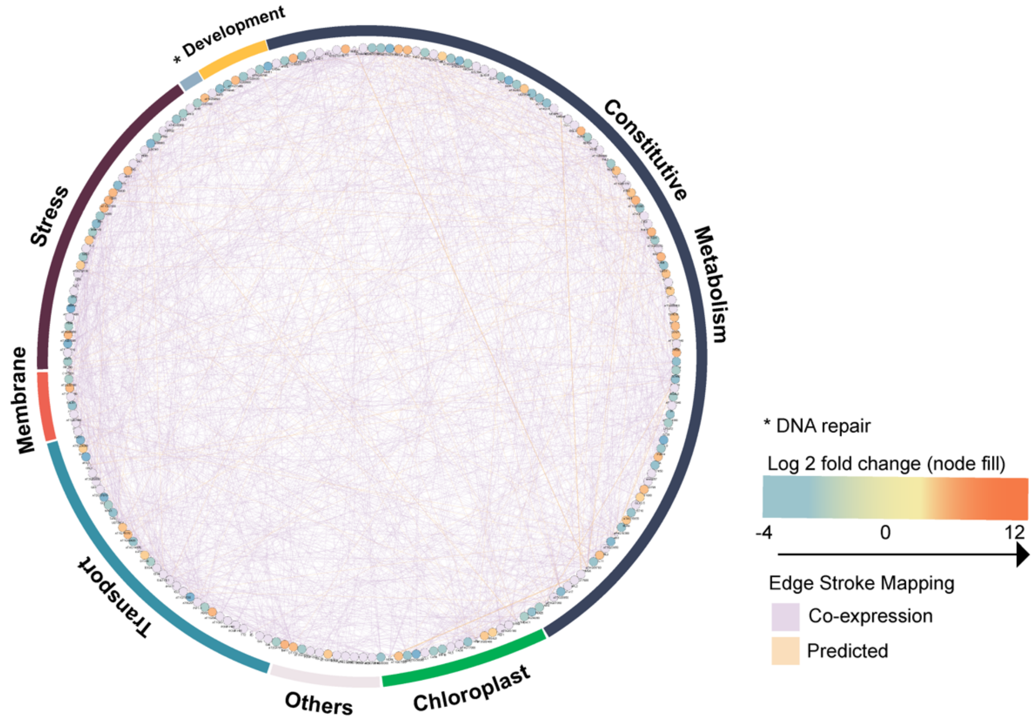

Figure S4. Interaction network for *P. albicaulis* gene interaction classification. Up-regulated on orange circles and down-regulated on purple circles in the inoculated samples, dividing genes accordingly to functionality according to KEGG ontology terms. Gray circles interactors genes viewed with GeneMANIA-related genes, crossing lines yielding co-expression interactions (purple), and shared protein domains (teal).

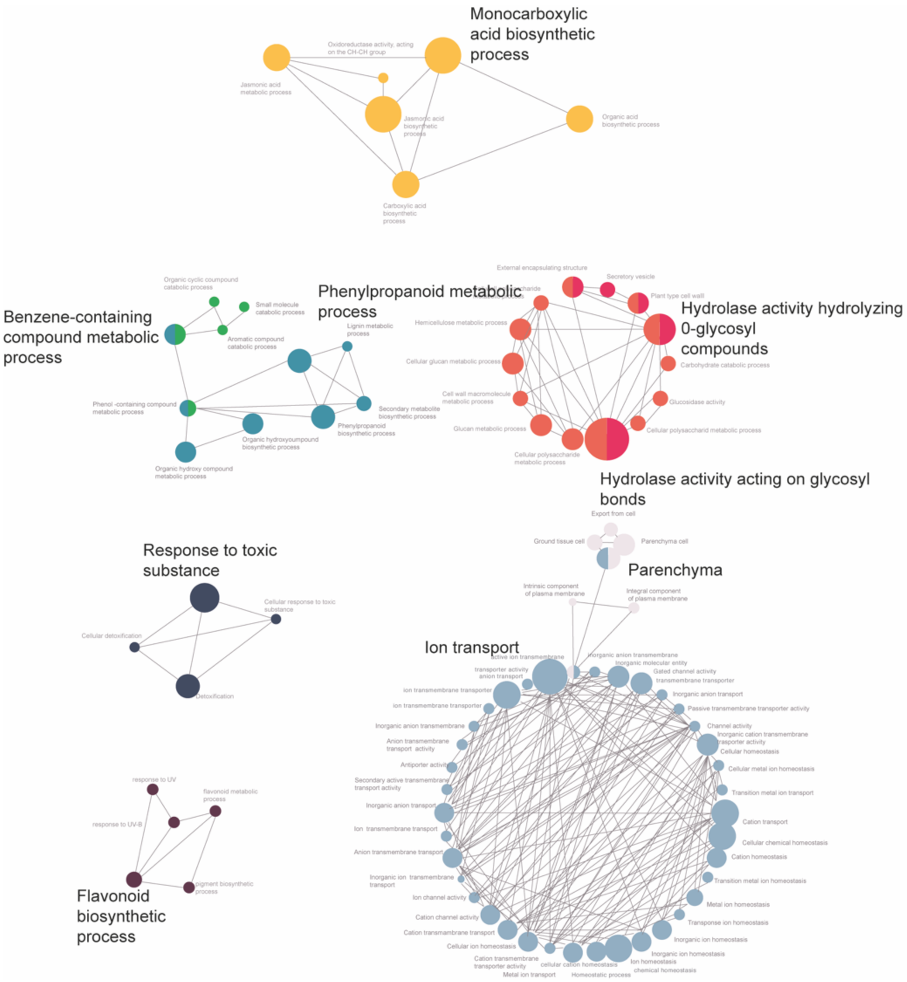

Figure S5. Gene interaction based the ortholog classification of DE gene families associated with abiotic response

Supplementary tables

Table S1. Statistics number of reads per sample and aligned to the reference transcriptome for each sample

		Total number of reads Sequenced (M)	Total number of reads Trimmed (M)	MAPPED READS (%)
Inoculated	1281	21.2	21.1	71.59
	1282	23.5	23.4	72.65
	1283	24	23.8	68.9
Non-Inoculated	1284	21.9	21.3	73.03
	1285	24.6	24.4	71.15
	1286	19.7	19.5	70.79

Table S2. Summary statistics for *de novo* assembly

	Inoculated			Non- Inoculated			Assemble ORP
**Assembly TRINITY**	1281	1282	1283	1284	1285	1286	Merged
Transcripts	39112	40849	39405	34763	43704	40301	139837
N50	2605	1297	2759	505	1682	2908	2882
Transcripts (after usearch)	45594	47635	44799	39359	52025	44891	86369
N50	2250	321	498	939	1764	774	726
**BUSCOs (%)**							
Complete	60.40	73.60	61.70	59.70	79.10	60.90	88.50
Single-copy	39.50	49.40	41.50	42.20	50.70	38.50	46.60
Duplicated	20.90	24.20	20.20	17.50	28.40	22.40	41.90
Fragmented	12.40	8.70	12.60	12.70	6.40	12.30	2.90
Missing	27.20	17.70	25.70	27.60	14.50	26.80	8.60

**Table S3.** Contaminants detected based on similarly search annotation						
	Inoculated			Non-inoculated		
Individual	1281	1282	1283	1284	1285	1286
Total unique contaminants:	149 (0.94)	130 (0.86)	109 (0.75)	110 (0.81)	94 (0.58)	136(0.97)
Bacteria	2 (1.34)	3 (2.31)	2 (1.83)	1 (0.91)	0	1 (0.74)
Insecta	70 (46.98)	48 (36.92)	57 (52.29)	61 (55.45)	44 (46.81)	80 (58.82)
Fungi	77 (51.68)	79 (60.77)	50 (45.87)	48 (43.64)	50 (53.19)	55 (40.44)
Top 5 contaminants by species:	*Rhodotorula graminis* 4(2.68%) *Acyrthosiphon pisum* 4(2.68%) *Diaphorina citri* 3(2.01%) *Batrachochytrium dendrobatidis* 3(2.01%) *Agrilus planipenni* 3(2.01%)	*Sclerotinia sclerotiorum* 7(5.38%) *Botrytis cinerea* (3.85%) *Acyrthosiphon pisum* 3(2.31%) *Diaphorina citri* 3(2.31%) *Rhizophagus irregularis* 3(2.31%)	*Diabrotica virgifera* 4(3.67%) *Rhagoletis zephyria* 3(2.75%) *Aphis gossypii* 3(2.75%) *Diaphorina citri* 3(2.75%) *Galleria mellonella* 3(2.75%)	*Diabrotica virgifera* 6(5.45%) *Diaphorina citri* 4(3.64%) *Spodoptera litura* 3(2.73%) *Galleria mellonella* 3(2.73%) *Aspergillus melleus* 3(2.73%)	*Nilaparvata lugens* 4(4.26%) *Diaphorina citri* 4(4.26%) *Acyrthosiphon pisum* 3(3.19%) *Homalodisca vitripennis* 3(3.19%) *Galleria mellonella* 3(3.19%)	*Diabrotica virgifera* 5(3.68%) *Nilaparvata lugens* 4(2.94%) *Pieris rapae* 3(2.21%) *Spodoptera litura* 3(2.21%) Bemisia tabaci 3(2.21%)

Table S4. Annotation statistics

EnTAP	Inoculated			Non- Inoculated			REF
	1281	1282	1283	1284	1285	1286	
Total Sequences	23926	24857	23604	21969	26085	23021	66233
N50	1218	1419	408	381	375	1203	2217
Similarity Search	14404	15159	14483	13545	16152	14044	43180
Total unique sequences with family assignment	18902	19387	18801	17840	20444	18800	56732
Total unique sequences with at least one GO term	16417	16579	16290	15436	17328	16427	48221
Total unique sequences with at least one pathway (KEGG) assignment	5243	5099	5217	4928	5169	5358	14165
Total unique sequences annotated (gene family and/or similarity search)	18917	19411	18818	17854	20465	18817	56796

Table S5. Transcriptome details of White pine species with transcriptomic evidence of infection of WBPR used in this study

Assembly TRINITY	*P. lambertiana*	*P. monticola*	*P. albicaulis* Reference
Transcripts	43194	81294	139837
N50	1648	4940	2882
Transcripts2	48544	65329	86369
N50	699	3846	726
BUSCOs (%)			
Complete	60.1	75.2	88.50
Single-copy	49.4	58.4	46.60
Duplicated	10.7	16.8	41.90
Fragmented	12.8	8.7	2.90
Missing	27.1	16.1	8.60
